# Correlates of Protective Motivation Theory (PMT) to Adolescents’ Drug Use Intention

**DOI:** 10.3390/ijerph110100671

**Published:** 2014-01-03

**Authors:** Cynthia Sau Ting Wu, Ho Ting Wong, Lai Yan Chou, Bobby Pak Wai To, Wai Lok Lee, Alice Yuen Loke

**Affiliations:** 1 School of Nursing, the Hong Kong Polytechnic University, Hong Kong, China; E-Mails: frank.ht.wong@polyu.edu.hk (H.T.W.); alice.yuen.loke@polyu.edu.hk (A.Y.L.); 2 Tuen Mun Hospital, Hong Kong, China; E-Mail: yycharlene@yahoo.com.hk; 3 Alice Ho Ming Ling Nethersole Hospital, Hong Kong, China; E-Mail: bobby_0405@yahoo.com.hk; 4 Queen Mary Hospital, Hong Kong, China; E-Mail: dreamtheater_1921@hotmail.com

**Keywords:** Chinese adolescents, drug use intention, protective motivation theory

## Abstract

Early onset and increasing proliferation of illicit adolescent drug-use poses a global health concern. This study aimed to examine the correlation between Protective Motivation Theory (PMT) measures and the intention to use drugs among adolescents. An exploratory quantitative correlation design and convenience sampling were adopted. A total of 318 students completed a self-reported questionnaire that solicited information related to their demographics and activities, measures of threat appraisal and coping appraisal, and the intention to use drugs. Logistic regression analysis showed that intrinsic and extrinsic rewards were significant predictors of intention. The odds ratios were equal to 2.90 (*p* < 0.05) and 8.04 (*p* < 0.001), respectively. The logistic regression model analysis resulted in a high Nagelkerke R^2^ of 0.49, which suggests that PMT related measures could be used in predicting drug use intention among adolescents. Further research should be conducted with non-school adolescents to confirm the application.

## 1. Introduction

The increasing usage of illicit drugs among adolescents is not only a burden on health and social-care expenditures, but it also causes wide-ranging social problems. Furthermore, drug abuse has tremendously negative effects on the physical, psychological and social well-being. In 2010, it was estimated that between 3.4% and 6.6% of the global adult population were users of illicit drugs [1]. In Hong Kong, the prevalence of drug-use among students was estimated to be 2.0% in 2009 [2].

The use of illicit drugs among adolescents could be attributed to the misconception that drug use falls within the normal parameters of society [3]. In connection with the increasing prevalence of illegal drugs, a receptive attitude regarding drug use could normalize drug misuse behavior [4]. Adolescents often share similar values with their peers and friends; thus, peer influence and normalization can overcome the consequences of drug use. Adolescents might also overlook the illegality of taking drugs [5,6], possibly because they rarely witness drug users being captured by the police [7]. The resulting perception is that the police cannot effectively tackle drug problems, which cause issues by spreading among peers or being observed by adolescents who previously hesitated to use drugs.

Shek [3] suggested systemic drug education was a tactic for dealing with the curiosity of adolescents, and systemic youth programs were recommended in counteracting undesirable peer influence. The suggestion remained unclear about the content that should be used in a systemic intervention approach. However, preventive measures could be particularly effective at schools, as they are widely recognized as having a significant influence on both the health and social well-being of students [8].

Since 2007, the Hong Kong government has been increasing funding to support various anti-drug programs. These programs were organized by education institutions, non-government organizations and hospitals [2]. Moreover, the Narcotic Council of Hong Kong (NCHK) has been offering cross-curricular drug education programs to schools since 2006. Drug-related topics have been incorporated at the secondary school level into subjects like “Personal, Social and Humanities Education”. The programs focused on the consequences of drug abuse, psychosocial support and refusal techniques aimed at preventing drug misuse among adolescents [9].

**Figure 1:**
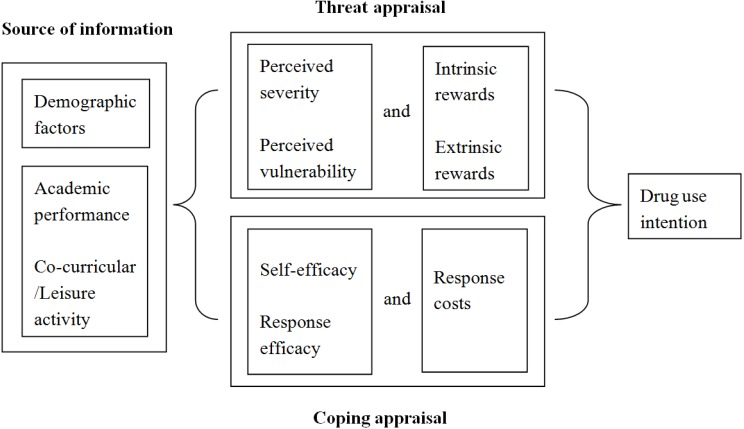
Protective Motivation Theory.

The reported cases of drug use among adolescents continue to increase, despite the efforts of drug education in schools. Limited local research studies were conducted to identify factors that could more effectively prevent adolescents from starting to use drugs. This study aimed to use Rogers’ Protective Motivation Theory (PMT) to test a framework for drug use intention among adolescents [10]. While similar theories like the social cognitive theory are frequently used to assess addictive behaviors and focus on aspects like self-efficacy, the PMT focuses on relevant cognitions [11]. It is a unique social cognitive model that prioritizes relevant cognitions and attempts to predict health-related behaviors and outcomes [12]. Researchers have applied the theory to different areas related to adolescents, such as intention to drink and drive [13], physical activity [14], smoking [15,16], and drug trafficking [17]. Because of PMT’s uniqueness and its previous successful application to the problem of drug trafficking, it was chosen for this study. A diagram summarizing the PMT model in relation to drug use intention can be found in Figure 1. 

The differences of the appraisal factors between two groups—one with the intention to use drugs (intention group) and one without it (no-intention group)—will be tested, and the corresponding predicting factors will be identified. The findings in this study could contribute to the development of a scientific framework to assess drug usage intentions among adolescents, and help validate strategies for drug use intervention.

## 2. Methods

### 2.1. Study Design

A quantitative cross-sectional exploratory design using convenience sampling was adopted. This study aimed to examine the relationship between adolescents’ drug use intention, demographic factors and the protection motivation level. Invitation letters were emailed to twenty-four secondary schools, selected randomly from a list of public schools. Three schools expressed their interest in joining after one month. The project team talked to the teachers and principals in face to face meetings, and before the study was conducted, written information about the research and the nature of the students’ involvement was sent to the participating students and their parents for their reference. Questionnaires were distributed to students in class, without teachers being present so there was no fear of unnecessary data exposure. The students needed around thirty minutes to complete the questionnaires in the classroom. Completed questionnaires were dropped into a sealed box by the students.

Ethics approval for this study was obtained from the ethics committee of the Hong Kong Polytechnic University (HSEARS20090826004). Informed consent was obtained from all participants. Students received a verbal explanation about the study aim, objectives, their right of withdrawal and confidentiality, before the questionnaire was distributed. 

### 2.2. Instrument

The questionnaire was self-reported and consisted of two parts; the first contained 22 questions based on the PMT [17], and the second focused on general information, including demographics. The PMT questions were measured on a five-point Likert scale. Seven factors were measured, namely perceived severity, perceived vulnerability, intrinsic rewards, extrinsic rewards, response cost, response efficacy, and self-efficacy. The higher the severity score, the more severe subjects perceived harm related to drug use was. Similarly, higher vulnerability scores expressed their perceived probability to experience harmful consequences of drug use. To illustrate the factors, examples like “*Kids who use drugs will get hurt*” and “*People who use drugs become addicts*” were used for perceived severity and vulnerability, respectively. The factors of intrinsic and extrinsic rewards expressed the perceived reinforcement of starting or continuing to use drugs. “*Taking drugs makes me feel away from the troubles*” is an example for intrinsic rewards, while “*I would know more friends if I tried drugs*” is an example for extrinsic rewards. Response cost indicated subjects expecting to lose peer respect and peer acceptance due to drug use. The higher the score, the higher the expectation of losing peer respect and peer acceptance. Example sentences like “*My friends would look down on me if I used drugs*” were used. Higher scores in response efficacy represented a higher level of self-ability to respond correctly to avoid drug use, and higher self-efficacy referred to higher self-ability to avoid the temptation of drug use. “*If my friends started using drugs, I would not hang out with them anymore*” and “*I could refuse to use drugs even if my friends were using drugs*” are examples for response efficacy and self-efficacy, respectively. In this study, all items were randomized to minimize the consistency effect [18].

The general information section of the questionnaire contained seven questions on demographic data in dichotomous and multiple choice response formats. They covered age, gender, religion, living with parents, type of housing, monthly pocket money, monthly disposable household income. One question dealt with school performance, two questions with time spent on extra-curricular activity and use of leisure or free time, and one question on the intention to drug use within the last twelve months. The questionnaire was reviewed by three experts from the fields of health care, research and adolescent health. A content validity index of 0.969 was obtained. Twenty eligible adolescents were invited to conduct test-retest reliability in a two week interval. The average correlation coefficient, calculated using Spearman’s rank correlation coefficient, was 0.817.

### 2.3. Data Analysis

The basic demographic characteristics of the participants were analyzed using descriptive statistics. The chi-square test/Fisher exact test and the Mann-Whitney U test were used to compare the intention and no-intention groups by demographic factors and PMT measures. Demographic factors with a *p*-value of less than 0.1 were further related to PMT measures by using binary logistic regression; the aim was to identify predictors for drug use intention. SPSS 18 was used for all statistical analysis, and statistical significance was set to 0.05.

## 3. Results

### 3.1. Demographic Factors, Academic Performance and Activity

The sample consisted of 318 school students. Nearly half of them (151, 47.5%) were between 13 and 15 years, and slightly more than half were male (183, 57.5%). The majority had no religious beliefs (211, 66.7%) and lived with both parents (242, 76.1%). Most of the students (127, 39.9%) received $HK 150 or less pocket money per month, and nearly a quarter (78, 24.5%) had $HK 501 or more available to spend. Out of the 194 (61.0%) students that their school provided an academic reference for, 79 (40.7%) ranked their academic performance in the upper half of the class. The majority spent six hours or less (234, 73.6%) with extracurricular activities per week, and 71.4% (227) took part in leisure activities. A summary of the demographic characteristics can be found in Table 1.

### 3.2. Drug Use Intention within the Last Twelve Months

Overall, 5.0% (16) of the respondents reported having had the intention to use illegal drugs within the last twelve months. Results of the Chi-square test/Fisher exact test showed that the factors of gender, family structure and pocket money were significantly different among students with and without drug use intention. Results indicated that a significantly higher proportion of female students had the intention to use drugs (male: 2.7%, female: 8.1%, *p* < 0.05). Students living with both parents tended to have a lower intention to use drugs than those living with one parent or those not living with parents (living with both parents: 3.3%, one parent: 10.0%, not living with parents: 12.5%, *p* < 0.05). Students who had more than $HK 501 available per month were found to have a higher intention to use drugs (above $HK 501: 11.5%, $HK 150–500: 2.7%, below $HK 150: 3.1%, *p* < 0.05) (Table 1).

**Table 1:** Demographic Characteristics and Drug Use Intention (N = 318).

Demographic Characteristics		With Drug Use Intention (n = 16)	Without Drug Use intention (n = 302)	Overall (N = 318)	χ^2^
		*n* (%)	*n* (%)	*n* (%) ^#^	
**Age**	*≤12*	1 (1.5)	67 (98.5)	68 (21.4)	4.97
	*13–15*	6 (4.0)	145 (96.0)	151 (47.5)	
	*16–21*	9 (9.1)	90 (90.9)	99 (31.1)	
**Gender**	*Male*	5 (2.7)	178 (97.3)	183 (57.5)	4.77 *
	*Female*	11 (8.1)	124 (91.9)	135 (42.5)	
**Religion**	*Yes*	6 (5.7)	100 (94.3)	106 (33.3)	0.13
	*No*	10 (4.7)	201 (95.3)	211(66.7)	
**Family structure**	*Living with both parents*	8 (3.3)	234 (96.7)	242 (76.1)	6.68 *
	*Living with one of the parents*	6 (10.0)	54 (90.0)	60 (18.9)	
	*Not living with parents*	2 (12.5)	14 (87.5)	16 (5.0)	
**Type of housing**	*Public rental*	2 (2.0)	98 (98.0)	100 (31.4)	3.52
	*Home ownership scheme*	4 (5.9)	64 (94.1)	68 (21.4)	
	*Private*	7 (6.3)	105 (93.8)	112 (35.2)	
	*Others*	3 (7.9)	35 (92.1)	38 (11.9)	
**Monthly household income**	*≤$10*,*000*	2 (6.3)	30 (93.8)	32 (10.0)	4.05
	*$10*,*001–$25*,*000*	5 (7.8)	59 (92.2)	64 (20.1)	
	*$25*,*001–$50*,*000*	2 (9.1)	20 (90.9)	22 (6.9)	
	*≥$50*,*001*	1 (5.3)	18 (94.7)	19 (6.0)	
	*Don*’*t know*	6 (3.3)	175 (96.7)	181 (56.9)	
**Pocket money**	*$0–$150*	4 (3.1)	123 (96.9)	127 (39.9)	7.63 *
	*$151–$500*	3 (2.7)	110 (97.3)	113 (35.5)	
	*≥$501*	9 (11.5)	69 (88.5)	78 (24.5)	
**Academic performance**	*Upper 25%*	0 (0.0)	31 (100)	31 (9.7)	5.27
	*26%–50%*	1 (2.1)	47 (97.9)	48 (15.1)	
	*51%–75%*	7 (9.2)	69 (90.8)	76 (23.9)	
	*Lower 24%*	3 (7.7)	36 (92.3)	39 (12.3)	
	*Not provided by school*	5 (4.0)	119 (96.0)	124 (39.0)	
**Extra-curricular activity (h)**	*0–2*	4 (4.0)	97 (96.0)	101 (31.8)	1.86
	*2<* *hour ≤6*	7 (5.3)	126 (94.7)	133 (41.8)	
	*6<* *hour ≤12*	4 (8.7)	42 (91.3)	46 (14.5)	
	*>12*	1 (2.6)	37 (97.4)	38 (11.9)	
**Use of leisure/** **Free time**	*Yes*	11 (4.8)	216 (95.2)	227 (71.4)	0.06
	*No*	5 (5.5)	86 (94.5)	91 (28.6)	

Notes: * *p < 0.05*; ^#^ Column percentage*.*

### 3.3. Protection Motivation Measure and Drug Use Intention

#### 3.3.1. Threat Appraisal

Table 2 shows that the mean score for threat appraisal was significantly lower in respondents with drug use intention than it was for those with no-intention (3.31 *vs.* 4.13, *p* < 0.001). Subjects in the intention group reported 3.63 and 3.85 for perceived severity and vulnerability, while subjects in the no-intention group reported 4.30 and 4.17. Although subjects with drug use intention had lower scores, only the difference in perceived severity was statistically significant (*p* < 0.05). Subjects with drug use intention reported higher intrinsic and extrinsic rewards of 3.04 and 3.19, while subjects without intention indicated scores of 1.80 and 2.14 (*p* < 0.001). (Table 2)

#### 3.3.2. Coping Appraisal

The mean score for coping appraisal in the intention group (3.24) was significantly lower than in no-intention group (3.60) (*p* < 0.05). The former also reported 3.13 and 2.96 in the categories of response efficacy and self-efficacy (no-intention: 3.70 and 3.61). The different responses to efficacy and self-efficacy were statistically significant with p-values smaller than 0.05 and 0.001, respectively. The intention group also reported a higher response cost of 3.63 *versus* 3.50 in the no-intention group, but the difference was not significant (Table 2).

**Table 2:** PMT Measures and Drug Use Intention (N=318).

	With Drug Use Intention	Without Drug Use Intention	*z*-value
	Mean (SD)	Mean (SD)	
**Threat appraisal ^#^**	3.31 (0.79)	4.13 (0.50)	4.47 ***
Perceived severity	3.63 (1.22)	4.30 (0.73)	2.27 *
Perceived vulnerability	3.85 (1.02)	4.17 (0.67)	1.30
Intrinsic rewards	3.04 (1.09)	1.80 (0.77)	4.47 ***
Extrinsic rewards	3.19 (0.75)	2.14 (0.56)	5.20 ***
**Coping appraisal**	3.24 (0.73)	3.60 (0.46)	2.21 *
Response costs	3.63 (0.67)	3.50 (0.62)	0.51
Response efficacy	3.13 (1.07)	3.70 (0.73)	2.00 *
Self-efficacy	2.96 (0.81)	3.61 (0.58)	3.55 ***

Notes: ^#^ The scores of intrinsic and extrinsic rewards were flipped before calculating the threat appraisal score; * *p* < 0.05; ** *p* < 0.01; *** *p* < 0.001.

#### 3.3.3. PMT Predictors of Drug Use Intention

Results of the logistic regression analysis showed that intrinsic and extrinsic rewards were significant predictors of students’ drug use intention. The corresponding odds ratios were 2.90 (95% CI = 1.24 − 6.81, *p* < 0.05), and 8.04 (95% CI = 2.63 − 24.56, *p* < 0.001). This means a one unit increase in the score of intrinsic/extrinsic reward increased the odds of drug use intention by a factor of 2.90/8.04. The regression model's Nagelkerke R^2^ was 0.49, indicating that 49% of the variation in the outcome variable can be explained by the model, which can be considered as high in the field of social sciences (Table 3). In addition, both the Hosmer-Lemeshow goodness of fit test and Omnibus test of model coefficients showed that the regression model was valid. Particularly, the Hosmer-Lemeshow goodness of fit test resulted in a chi-square value of 3.67 (*p* = 0.886), meaning that the observed and predicted probabilities matched. The Omnibus test of model coefficients gave a chi-square value of 55.39 (*p* < 0.001), showing that the proposed model was significantly better than the model without any predictors.

**Table 3:** PMT Predictors of Drug Use Intention (N = 318).

PMT Variables	Odds Ratio	95% CI	Nagelkerke R^2^	Hosmer and Lemeshow Test (χ^2^)	Omnibus Tests of Model Coefficients (χ^2^)
Perceived severity	0.55	(0.18, 1.67)	0.49	3.67	55.39 ***
Perceived vulnerability	1.70	(0.48, 6.01)			
Intrinsic rewards	2.90 *	(1.24, 6.81)			
Extrinsic rewards	8.04 ***	(2.63, 24.56)			
Response costs	1.71	(0.53, 5.50)			
Response efficacy	0.99	(2.97, 3.30)			
Self-efficacy	1.22	(0.35, 4.25)			

Notes: + Method = Enter; ^#^ Controlled demographic variable: Gender; * *p* < 0.05; ** *p* < 0.01; *** *p* < 0.001.

## 4. Discussion

### 4.1. Overall Prevalence

In this study, 5.0 % of the respondents indicated they had the intention to use drugs, which is a slightly higher value than the 4.3% that was reported by the NCHK [19]. The difference is acceptable because it could be due to factors like the year, setting, sample size or random error.

### 4.2. Age

The probability for adolescents to form strong connections with their peers increases with age. Research suggests that major transitions in the lives of adolescents, like changes in physical development or social situations, increase the chance they use drugs [20]. Secondary students who are aged from 13 to15 often experience new school regulations and are exposed to increased academic and peer pressure. Newman established a revision of Erikson’s model of psychosocial stages [21] and suggests that among the eleven psychosocial developmental stages they identified, adolescents in the ages from 12 to 18 were having a psychosocial crisis of ‘group identity *versus* alienation’ and a developmental task of ‘membership in peer group’. The result of this study also showed the prevalence of the intent to drugs increased with age, although the increase was statistically insignificant.

### 4.3. Gender

There were a higher percentage of female students in the intention group. This is in contradiction with recent findings indicating that a higher percentage of males are using drugs [22]. This gender difference might be attributed to a change of sex-role expectations; females and males share similar education and job opportunities. Females are less expressive and more goal-orientated, which may lead to maladaptive behavior [23]; further, a less expressive personality could discourage them to seek external help, and female adolescents may also underestimate their vulnerability toward drug use. On the other hand, the insignificant difference of the proportions of boys and girls living with single parents (boys: 18.6%, girls: 19.3%) suggested that the higher percentage of female students in the intention group was unlikely due to higher proportion of girls living with single parents. Moreover, the data resolution of this study was not high enough for finding out if the girls having an earlier age of completing puberty played a significant role in the prevalence of girls’ drug use intention.

### 4.4. Living with Parents

Studies found that in a household with both parents, more supervision and monitoring of children can be provided, leading less drug use [24,25]. Niaz *et al.* [26] also claimed that there were significant positive correlations between drug misuse and separated parents. The results in the present study are consistent with these findings; adolescents who lived in two-parent households reported a significantly lower intention to use drugs.

Given that adolescents have limited life experience, supervision and advice from parents are important. Adolescents who are not living with their parents can feel more vulnerable as they are not being physically and psychologically protected. They may also receive less parental guidance when dealing with difficult situations. Further, adolescents from households with single or no parents also lack parental role models in terms of refusing drug use. Lastly, parents can develop preventive actions and provide verbal persuasion to help with avoidance of undesirable behaviors.

### 4.5. Monthly Disposable Household Income

According to the result of the NCHK [22], drug-taking students were mostly from high and low income families. Researchers also found that adolescents raised in low income families by parents with long work hours and low wages received less supervision and care; this can result in children being more prone to developing drug use problems [23,27]. A different study suggested adolescents with high household incomes were associated with a higher rate of substance use [28]. However, over half of the participants in this study reported that they did not know their monthly household incomes, which could mask the actual influence household income has on drug use among adolescents.

### 4.6. Monthly Pocket Money

In the present study, monthly pocket money referred to the net savings, excluding travelling expenses and meals. Previous research showed that the main source of money for drug purchases was pocket money [22,29], and that higher amounts of pocket money were associated with greater risk of substance use [30]. The results of the present study are consistent with these findings; adolescents with a monthly pocket money in excess of $HK 501 had a significantly higher intention of drug use. However, determining an exact amount of pocket money that exposes adolescents to a higher risk of drug use is difficult, as the amount may vary due to different living standards and cultures in different societies.

### 4.7. Academic Performance

Studies have shown that substance use in adolescents was associated with decreased educational attainment [28,31]. Henry [32] also claimed that students may risk an escalation of their drug use if there was a decline in academic achievement. Undesirable academic performance may lower the self-efficacy of adolescents and lead to negative emotions. Poor academic performers are also more likely to associate with deviant peers, which would create a social structure that promotes substance use [33]. Adolescents could also develop drug use problems in an effort to gain recognition and acceptance within groups of deviant peers that are using drugs. This study came to similar results; students who claimed that their academic performance was ranked above average showed a significantly lower tendency to use drugs. 

### 4.8. Perceived Severity

The mean value for perceived severity was significantly higher in the no-intention group than the intention group. A recent NCHK report found that over 70% of adolescents reported they were afraid of the consequences of drug use, and approximately 95% of the non-drug taking group agreed that it would cause harm to one’s health; in the drug-taking group, nearly 85% agreed [22]. Peer effects could affect the perception of drug misuse; for example, adolescents could perceive issues related to drug use as less severe if their peers were drug users.

### 4.9. Extrinsic Rewards

In the present study, extrinsic rewards were significantly higher in the intention group. Lam and Wong [7] found that drug taking behavior was significantly influenced by peers, and was regarded as a way to obtain social inclusion. Increasing their social recognition and support can be reasons for adolescents to be susceptible to peer influence as well. Thus, undesirable peer influence is a risk factor for drug use and it may serve as an origin of perceived positive outcomes. Adolescents could also feel humiliated if they were isolated or shunned [5]. They care about peer recognition in their psychological and social development [34]. Overall, adolescents may be motivated to use drugs for the sake of wanting to be recognized by a group or as a person who is brave enough to try new things.

### 4.10. Intrinsic Rewards

Intrinsic rewards were higher in the intention group. The NCHK [22] and Niaz *et al.* [26] came to similar results, showing that secondary students took drugs to satisfy curiosity and to relieve boredom. Adolescents are curious about new things and ideas; they are adventurous and sometimes impulsive [34]. Their values are still in the process of shaping. They face challenges and difficulties in dealing with relationships, which can be daunting when their decision making skills are inadequate [35]. Adolescents tend to use psychoactive substances when encountering stress, boredom and harsh reality. They might treat drug use as a means of emotional self-regulation or a way to escape from overwhelming situations [4,26,35]. Elevation of intrinsic rewards may occur especially when adolescents witness friends experiencing positive emotions when taking drugs. The excitements from taking drugs can temporarily free from troubles and motivate them to use drugs themselves.

### 4.11. Self-Efficacy

Self-efficacy can be regarded as a form of self-confidence. Self-efficacy was higher in the no-intention group than it was in the intention group. Non-drug taking students believed that they could manage difficult problems if they tried hard [22]. High self-efficacy implies a strong belief in the ability and control of vicarious experience in protecting oneself against drug use, and not being easily convinced. As a result, they were prone to less drug use intention.

### 4.12. Response Efficacy

Response efficacy was higher in participants without drug use intention. Self-efficacy is affected by vicarious experiences and verbal persuasion [36]. Experience sharing from past-drug users augments self-efficacy, and a successful experience would boost one’s faith to follow certain actions to refuse drug use. Adolescents receive information from trusted people, such as peers, teachers, family, and experienced people. The involvement of this group of people may increase the effectiveness of the actions to refuse drug use.

### 4.13. Predictors of Drug Use Intention

The logistic regression showed that a very good regression model could be developed using PMT. This was demonstrated by the high Nagelkerke R^2^ and the results of the goodness of fit tests. Particularly, the two significant predictors, intrinsic and extrinsic rewards, could serve as a guide for health educators and help design more focused and evidence-based drug abuse prevention campaigns in school settings. As the factors of perceived severity and vulnerability were not significant predictors, this suggests that emphasizing the harmful effects of drug abuse on youths could be less effective. Instead, the role of intrinsic and extrinsic rewards could be emphasized.

### 4.14. Limitation

The sample only included adolescents who attended schools; non-school adolescents could be included to improve the representation of the sample. The subjects were only recruited in Hong Kong, meaning that the generalizability of the findings to Chinese adolescents from other cities may be limited. Moreover, the prevalence of drug use intention in this study may not accurately reflect the situation of actual drug abuse and misuse, in which the predictors may not be the same. Finally, the limited number of students who showed an intention to use drugs could undermine the model validity. Future research could increase the sample size and use a more sophisticated design to minimize the above limitations; for example using mixed method for further validating the results through conducing focus groups with different related parties. 

## 5. Conclusions

In this study, we developed a logistics regression model for explaining the relationship between PMT related measures and adolescents’ drug use intention. The threat appraisal outcome—particularly the intrinsic and extrinsic rewards in motivating adolescents’ response to protect self from drug misuse—may prove interesting for health-care professionals. The significant predictors of drug use intention provided hints for health educators to developed more focused drug misuse prevention programs in a right direction. The cost-effectiveness of drug misuse prevention programs could be improved by referencing to the results of this study.
